# Novel Surgical Treatment for Refractory Heel Ulcers in Werner's Syndrome

**DOI:** 10.1155/2013/287025

**Published:** 2013-08-26

**Authors:** Keisuke Oe, Masahiko Miwa, Yoshitada Sakai, Masahiro Kurosaka

**Affiliations:** ^1^Department of Orthopedic Surgery, Kobe University Graduate School of Medicine, 7-5-2 Kusunoki-cho, Chuo-ku, Kobe Hyogo 650-0017, Japan; ^2^Division of Rehabilitaion Medicine, Kobe University Graduate School of Medicine, 7-5-2 Kusunoki-cho, Chuo-ku, Kobe Hyogo 650-0017, Japan

## Abstract

Patients with Werner's syndrome frequently develop chronic leg ulcers that heal poorly. We present a patient who suffered from this rare syndrome and developed typical heel ulcers. Treatment of the ulcer is challenging, as flap options are limited over the lower third of the leg and skin grafting is not easy as there is a lack of healthy granulations. We successfully treated the ulcer with osteomyelitis by drilling the bone and applying an ultrathin split thickness skin graft with the thigh skin as the donor site.

## 1. Introduction

Werner's syndrome, first described in 1904, is believed to be an autosomal recessive inherited disorder [1]. The clinical findings include shortness of stature, premature graying and loss of hair, cataracts, atrophy and hyperkeratosis of the skin, changes in the timber of the voice, tendency to diabetes mellitus, arteriosclerosis, osteoporosis, and a high incidence of neoplasms [2].

Intractable ulcers are a big problem for patients of Werner's syndrome. The ulcers, occurring on the extremities, especially on elbow, patella, and heel regions, are resistant to conservative treatment. However, surgical treatments are also often unsuccessful because of a marked delay of wound healing, hard and inflexible skin, and arteriosclerotic changes, which are characteristics of Werner's syndrome.

We successfully treated an ulcer with osteomyelitis by drilling the bone and applying an ultrathin split thickness skin graft with the thigh skin as the donor site.

## 2. Case Report

**Figure 1:**
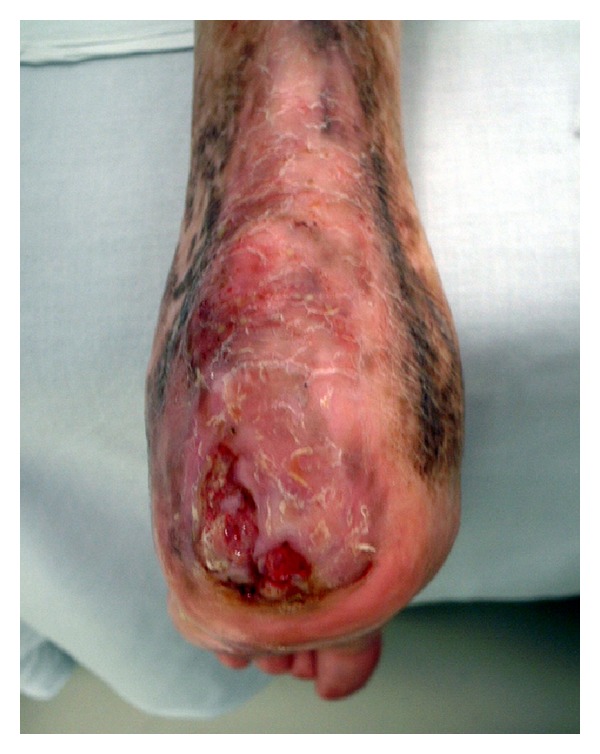
Chronic ulcer and exposed calcaneal cortex bone over the posterior aspect of the left heel.

**Figure 2:**
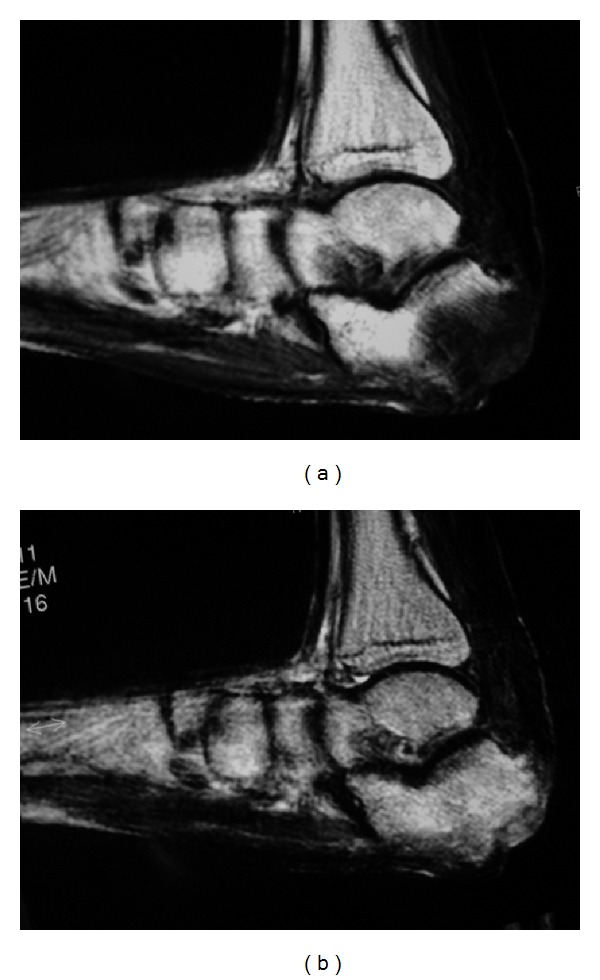
Magnetic resonance imaging of the preoperation. (a) T1-weighted sagittal imaging shows diffuse low signal intensity in posterior calcaneus. (b) T2-weighted sagittal imaging shows diffuse high signal intensity in the same region.

A 47-year-old man was admitted to the Department of Plastic Surgery, Kobe University Hospital, for treatment of a chronic ulcer on his left heel. The size of the ulcer was 4 × 3 cm, and the calcaneal cortex was partially exposed (Figure 1). There was no improvement in the ulceration following curettage and repeated local treatments for three months. Magnetic resonance imaging was performed. T1-weighted sagittal imaging showed diffuse low signal intensity in posterior calcaneus. T2-weighted sagittal imaging showed diffuse high signal intensity in the same region (Figures 2(a) and 2(b)). Based on these findings, osteomyelitis of the calcaneus was diagnosed, and the patient was referred to our department.

**Figure 3:**
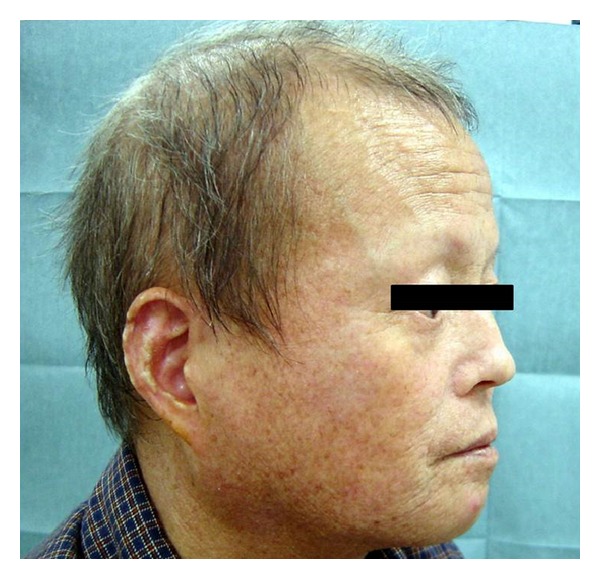
The typical bird-like facial characteristics of patient with Werner's syndrome.

General physical examination on admission showed that the patient was 165 cm in height and 54 kg in weight. His extremities were thin. Atrophied skin and decreased subcutaneous fat and muscle were observed in the periphery of the extremities. His external genitalia appeared atrophied. His hair was generally scarce and markedly gray. His face looked aged and had a “bird-like” appearance (Figure 3). His voice was high-pitched and hoarse. When he was 32 years old, he underwent surgery for cataracts in both eyes. 

His family history revealed that his parents were cousins, but there were no symptoms of note in his siblings, and no other family members had Werner's syndrome.

**Figure 4:**
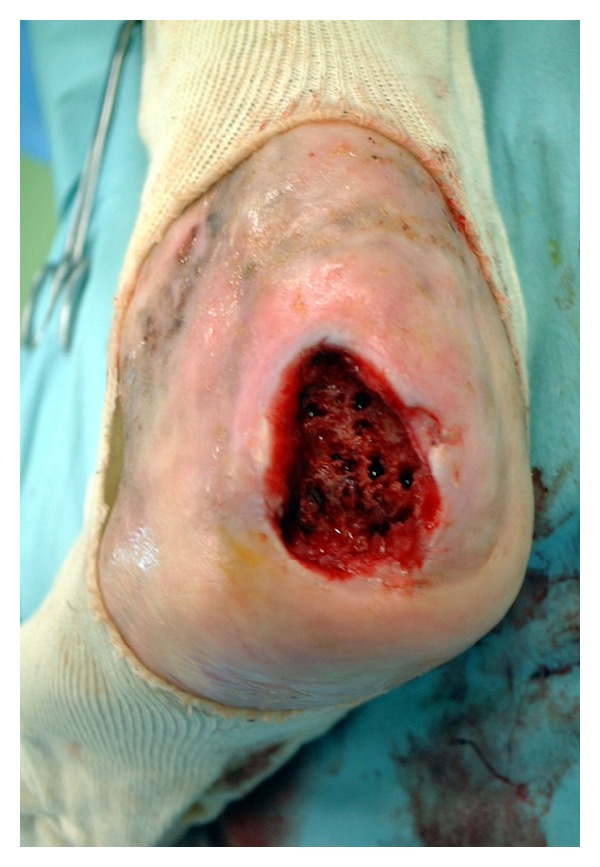
The ulcer received debridements and calcaneal bone was drilled manually by Kirschner wire.

**Figure 5:**
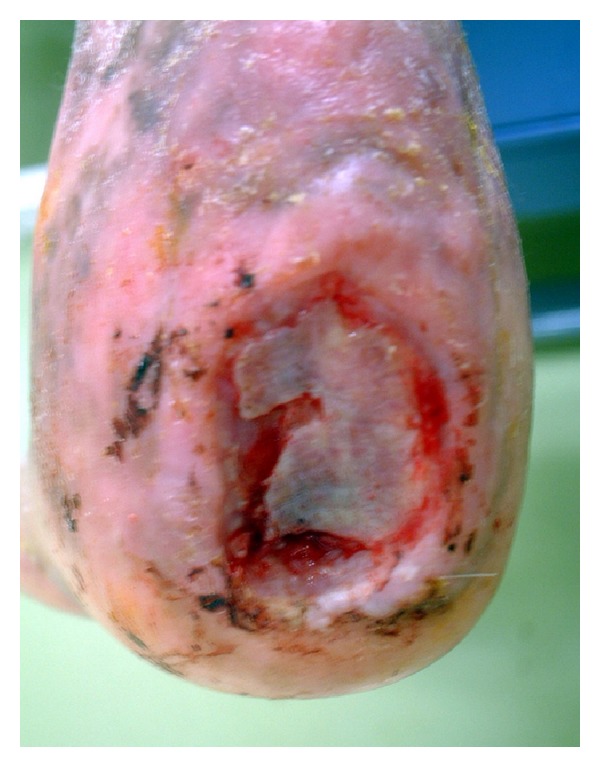
Five days after operation, ultra-thin split thickness covered this region.

**Figure 6:**
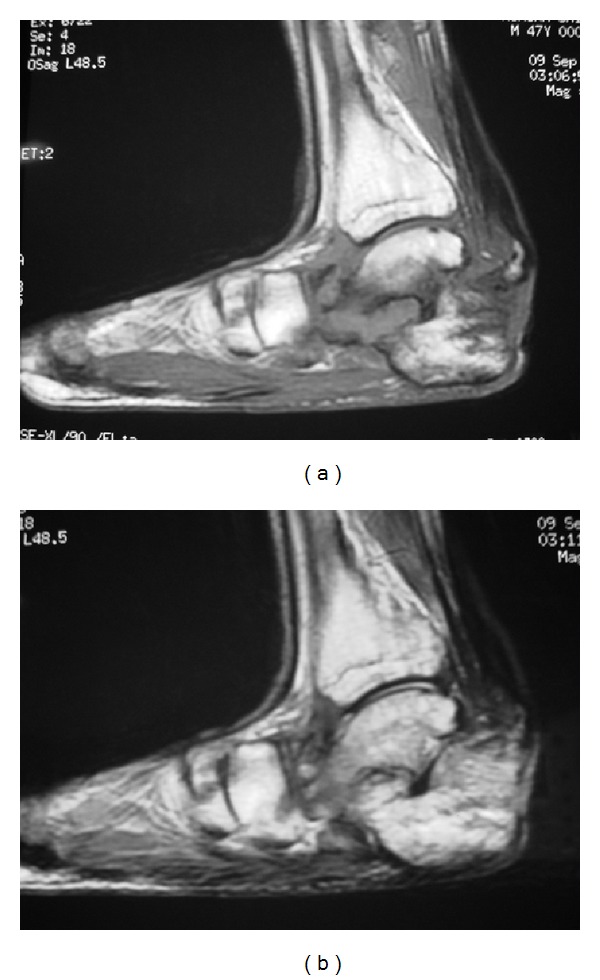
Magnetic resonance imaging of one year after operation. (a) T1-weighted sagittal imaging shows diffuse high signal intensity in posterior calcaneus. (b) T2-weighted sagittal imaging shows diffuse high signal intensity in the same region.

**Figure 7:**
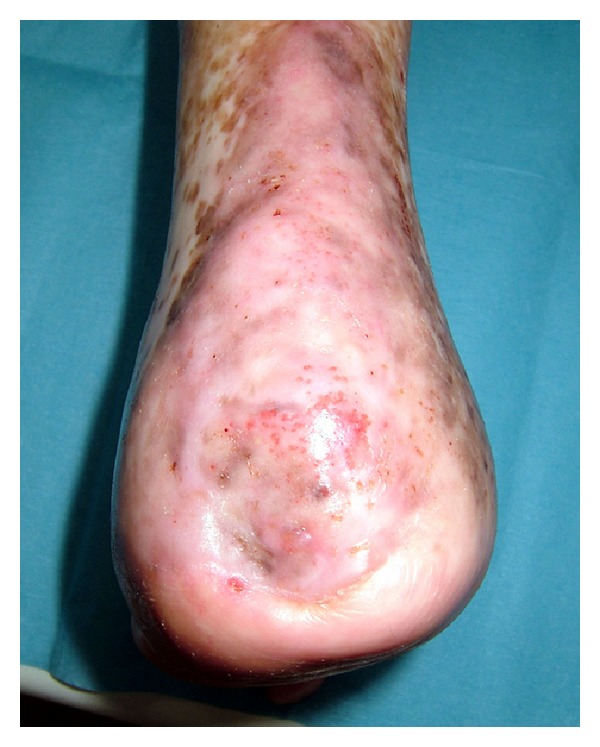
One year after operation, no recurrence of ulcers and osteomyelitis.

 Surgical treatment was considered because the ulcer did not respond to conservative treatment. Under general anesthesia, the ulcer was debrided and osteomyelitis was performed followed by a curettage of the dome form as fully as possible. We drilled the calcaneus in order to improve the recipient ground circulation (Figure 4). The drilling was performed manually with 1.5 mm diameter Kirschner wire (K-wire) at a number of center points, and with 1.0 mm in diameter K-wire at multiple periphery points. Five days after operation bleeding, of the calcaneus stopped and under local anesthetic the calcaneus was covered by an ultra-thin split thickness skin graft with the left thigh skin as the donor site (Figure 5). One month later, the lesion was healed. One year postoperatively, there has been no recurrence of the ulcer or the osteomyelitis (Figures 6(a), 6(b), and 7).

## 3. Discussion

Werner's syndrome was initially described by Werner in 1904 [1], when he reported four siblings with symptoms and signs including juvenile cataract, pachyderma-like alteration of the extremities, small stature, premature ageing of the face, juvenile grey hair, and genital hypoplasia. This unique condition was named Werner's syndrome by Oppenheimer and Kugel in 1934 [3]. About 1300 cases have been reported around the world from 1916 to 2002, and among them about 1000 cases have occurred among Japanese patients firmly establishing Japan as the country with the highest incidence of Werner's syndrome.

Table 1 lists the criteria for establishing the diagnosis of Werner's syndrome [4]. Our patient had all eight characteristic features of Werner's syndrome.

Treatment for heel ulcers in Werner's syndrome remains a challenging problem, as spontaneous wound closure is often difficult. The cause of refractory ulceration may include inflexible hard skin and disorders of the vasculature, such as arteriosclerotic changes in the blood vessels and decreases in local blood flow, extrinsic stimulation of the atrophic skin that has decreased ability to support the tissue, and complications of generalized metabolic disorders such as diabetes mellitus. Thus, poor healing capability of the refractory ulcer in patients with this disease is multifactorial. The frequency of early arteriosclerosis is about 50% in patients with Werner's syndrome [5]. Therefore, conservative treatments for heel ulcers including traditional wound dressings and hyperbaric oxygen therapy are usually not effective. Surgical procedures including rotational flaps, free flaps, and skin grafting have been reported [5, 6]. However, skin flaps pose the threat of irrecoverable damage to the circulation of the lower leg in the future, because arteriosclerosis is frequently present in Werner's syndrome patients due to diabetes mellitus. Therefore, this treatment should only be adopted after careful consideration especially as the patient's general condition may deteriorate if lengthy operations are performed. Skin grafting is not usually successful due to the exposure of tendons and bony structure or the lack of supporting granulation tissues. As a result, 20% of patients with Werner's syndrome with intractable ulcers subsequently undergo amputation of a lower limb [5].

In our procedure, we utilized multiple bone drilling to increase ground circulation and combined it with the use of an ultra-thin split thickness skin grafting, which is resistant to infections and adheres better than other thin split thickness skin grafts resulting in a complete resolution of symptoms.

To our knowledge, we could not find any other reports in the literature of bone drilling and ultra-thin split thickness skin grafting therapy adopted for the treatment of refractory ulceration associated with Werner's syndrome.

In conclusion, we reported the successful treatment of a patient with a chronic heel ulcer and osteomyelitis in Werner's syndrome. Our proposed surgical technique is both technically simpler and less invasive than other previously reported techniques [6, 7], and therefore we recommend its use for treatment of refractory ulceration as a treatment of choice for this difficult clinical problem.

## Figures and Tables

**Table 1:** Findings in Werner's syndrome.

Characteristic feature of Werner's syndrome	
(1) Arrest of growth at puberty	
(2) Development of senile cataracts in the third to fourth decade	
(3) Premature graying (canities) and balding	
(4) Scleroderma-like involvement of the extremities	
(5) Marked diminution of the muscle mass and subcutaneous tissue of the extremities	
(6) Chronic, slow-healing ulcerations over pressure points of the feet and ankles	
(7) Beak-shaped nose	
(8) Premature development of arteriosclerosis	

Additional frequent manifestations of Werner's syndrome	
(1) Impaired carbohydrate tolerance	
(2) Hypogonadism	
(3) Osteoporosis	
(4) Localized soft-tissue calcification	
(5) History of consanguinity and familial incidence, or both	
(6) Thin, high-pitched voice	
(7) Circumscribed hyperkeratosis	
(8) Multiple, recurrent, and painful callosities	
